# An ongoing search for potential targets and therapies for lethal sepsis

**DOI:** 10.1186/s40779-015-0047-0

**Published:** 2015-08-08

**Authors:** Guo-qiang Bao, Li He, David Lee, John D’Angelo, Hai-chao Wang

**Affiliations:** Department of Emergency Medicine, North Shore University Hospital, Manhasset, NY 11030 USA; The Feinstein Institute for Medical Research, 350 Community Drive, Manhasset, NY 11030 USA; Department of General Surgery, Tangdu Hospital, The 4th Military Medical University, Xi’an, Shaanxi 710032 China; Department of Ophthalmology, University of Alabama at Birmingham, Birmingham, AL 35294 USA

**Keywords:** Innate immune cells, Pathogen-associated molecular pattern molecules, High mobility group box 1, Herbal components, Sepsis, Autophagy, Endocytosis, Double-stranded RNA-activated protein kinase R

## Abstract

Sepsis, which refers to a systemic inflammatory response syndrome resulting from a microbial infection, represents the leading cause of death in intensive care units. The pathogenesis of sepsis remains poorly understood although it is attributable to dysregulated immune responses orchestrated by innate immune cells that are sequentially released early (e.g., tumor necrosis factor(TNF), interleukin-1(IL-1), and interferon-γ(IFN-γ)) and late (e.g., high mobility group box 1(HMGB1)) pro-inflammatory mediators. As a ubiquitous nuclear protein, HMGB1 can be passively released from pathologically damaged cells, thereby converging infection and injury on commonly dysregulated inflammatory responses. We review evidence that supports extracellular HMGB1 as a late mediator of inflammatory diseases and discuss the potential of several Chinese herbal components as HMGB1-targeting therapies. We propose that it is important to develop strategies for specifically attenuating injury-elicited inflammatory responses without compromising the infection-mediated innate immunity for the clinical management of sepsis and other inflammatory diseases.

## Introduction

Cohabitating with various microbes, mammals have developed multiple strategies for combatting microbial infections. As the first layer of defense, the epithelial barriers effectively limit the access and growth of the majority of pathogens. If they are breached, innate immune cells immediately launch biological responses, termed “inflammation,” to confine and remove these pathogens [1]. These inflammatory responses are usually appropriately propagated and often result in the successful elimination of the invading pathogens. If unsuccessful, the invading pathogens can leak into the bloodstream, triggering a widespread and systemic inflammatory response, termed “sepsis.” Sepsis, which refers to a systemic inflammatory response syndrome resulting from a microbial infection, represents the leading cause of death in intensive care units. As a continuum of increasing clinical severity, “severe sepsis” is often associated with one or more acute organ dysfunctions [2]. Despite recent advances in antibiotic therapy and intensive care, the overall mortality rate of severe sepsis remains high [3].

The inflammatory responses are first initiated by innate immune cells, such as macrophages and monocytes. These innate immune cells are equipped with pattern recognition receptors, such as the Toll-like receptors (TLRs), TLR2, TLR3, TLR4, and TLR9 [4–8], for various pathogen-associated molecular patterns (PAMPs), such as bacterial peptidoglycan, double-stranded RNA, endotoxin, and CpG-DNA [9, 10]. The engagement of various PAMPs with respective receptors triggers the sequential release of early (TNF, IL-1 and IFN-γ) and late (HMGB1) proinflammatory mediators [11–13]. Although early proinflammatory cytokines contribute to the pathogenesis of sepsis [14], their early kinetics of release makes them difficult to target in clinical settings.

### Discovery of HMGB1 as a late mediator of lethal sepsis

Approximately 20 years ago, we aimed to search for other late mediators that might contribute to the pathogenesis of lethal sepsis. To identify such mediators, we stimulated macrophages with early cytokines (e.g., TNF) and screened the cell-conditioned medium for proteins that were released late. The SDS-PAGE gel electrophoresis analysis revealed the release of 30-kDa protein with an N-terminal amino acid sequence identical to HMGB1 [13], a member of the high mobility group-1 non-histone chromosomal protein family.

#### Active HMGB1 Secretion

**Fig. 1:**
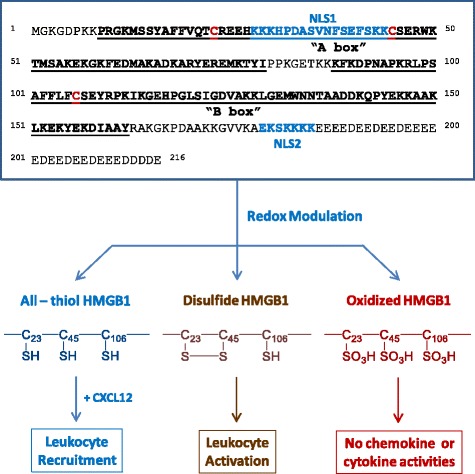
Redox modulation of HMGB1 immunological activities. The cysteine residues of HMGB1 can be divergently oxidized, which affects its chemokine or cytokine activities. Depending on the redox status, extracellular HMGB1 can either facilitate leukocyte recruitment or activation, resulting in rigorous inflammatory responses (cytokine storm) and organ dysfunction

HMGB1 is constitutively expressed to maintain a large “pool” of pre-formed protein in the majority of cells [15, 16]. Bearing two nuclear-localization sequences (NLS), HMGB1 is transported into the nucleus by the nuclear import complexes, thereby maintaining a large nuclear “pool” of pre-formed protein (Fig. 1) [17]. Within the nucleus, HMGB1 binds chromosomal DNA and fulfills its nuclear functions, such as maintaining the nucleosomal structure and regulating gene expression [18]. The disruption of local HMGB1 expression renders animals susceptible to infectious [19] or injurious insults [20, 21], indicating an overall beneficial role of intracellular HMGB1 [22]. In response to exogenous PAMPs (ds-RNA, CpG-DNA and endotoxin) [13, 23] or endogenous cytokines (interferon (IFN)-γ, IFN-β and cold-inducible RNA-binding protein (CIRP)) [24–26], macrophages/monocytes actively release HMGB1. If dysregulated, the excessive HMGB1 release adversely contributes to the pathogenesis of infection- and injury-elicited inflammatory diseases.

**Fig. 2:**
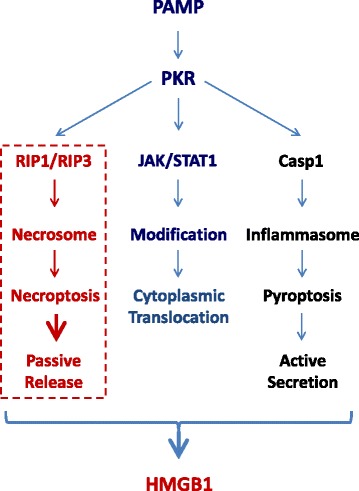
Essential roles of PKR in the regulation of HMGB1 release/secretion. HMGB1 is released by activated macrophages/monocytes through complex mechanisms dependent on the activation of PKR, which may regulate the JAK/STAT1-dependent nuclear-cytoplasmic HMGB1 translocation, RIP1/RIP3-dependent necroptosis, and caspase 1-dependent pyroptosis

Lacking a leader signal sequence, HMGB1 cannot be actively secreted via the classical ER-Golgi secretory pathway [13]. Instead, the activated macrophages/monocytes acetylate and phosphorylate HMGB1 at the nuclear localization or export sequences (NLS or NES) [22, 27–29] lead to the sequestration of HMGB1 within cytoplasmic vesicles, which are destined for secreting into the extracellular environment [16, 24, 30]. For instance, in response to exogenous PAMPs (e.g., endotoxin) or endogenous cytokines (e.g., IFNs), innate immune cells acetylate lysine residues 28, 29, 42, 43, 179, 181, and 183 within the NLS sites lead to the cytoplasmic HMGB1 translocation in a JAK/STAT1-dependent fashion (Fig. 2) [16, 24, 27, 30]. Indeed, the pharmacological inhibition or genetic interference with JAK/STAT1 signaling uniformly inhibits HMGB1 secretion induced by IFN-β, IFN-γ or LPS. Notably, LPS might not directly activate STAT1; however, it may trigger indirect STAT1 activation through the intermediate production of IFNs [31] that are capable of inducing HMGB1 release [24, 25, 27].

After cytoplasmic translocation, HMGB1 is secreted extracellularly through several pathways, including the caspase-1/caspase-11-mediated inflammasome activation and pyroptosis (Fig. 2). The pharmacological inhibition with a broad-spectrum caspase inhibitor (Z-VAD-FMK) or the genetic deletion of caspase-1/caspase-11 uniformly reduces HMGB1 secretion from activated macrophages [32, 33]. Similarly, the genetic disruption of the double-stranded RNA-activated protein kinase R (PKR) or the pharmacological inhibition of PKR phosphorylation similarly reduces NLRP3 or NLRP1 agonists-induced inflammasome activation [34, 35], pyroptosis [34, 35], and HMGB1 release [34]. In light of the likely roles of PKR in the regulation of caspase-1-dependent programmed cell death (pyroptosis) [35] and receptor interacting protein (RIP)1/RIP3-dependent programmed necrosis (necroptosis) (Fig. 2) [36], it is important to explore novel PKR inhibitors that may inhibit HMGB1 release by preventing distinct cell death pathways.

#### Passive HMGB1 Release

In addition to active secretion, HMGB1 can be passively released from damaged cells [37] after ischemia/reperfusion [38, 39], trauma [40, 41], or toxemia [42–44], thereby serving as damage-associated molecular pattern molecule (DAMP). Necrosis can also be induced by various viruses (e.g., West Nile, salmon anemia, dengue, and influenza viruses) [45, 46] and cytokines (e.g., TNF, IFNs) [36, 47]. Extracellular HMGB1 can also trigger caspase-1-dependent programmed cell death, pyroptosis, which is characterized by rapid plasma membrane rupture, and the release of proinflammatory intracellular contents (including HMGB1) [48], suggesting a pathogenic role of pyroptosis in HMGB1 release during infection or injury. Thus, infection and injury converge in a common process, i.e., inflammation [49], which is orchestrated by HMGB1 and other proinflammatory mediators (e.g., mitochondrial DNA and CIRP) released by activated immune cells and damaged tissues [26, 50].

Once actively secreted or passively released, extracellular HMGB1 binds to various microbial products (e.g., CpG-DNA or LPS), thereby facilitating their recognition by respective receptors to augment inflammatory responses [51]. Harboring three cysteine residues (C23, C45 and C106) that are redox-sensitive, HMGB1 can be modified into three isoforms termed “HMGB1” (all thiol form), “disulfide HMGB1” (partially oxidized), and oxidized HMGB1 (Fig. 1) [52, 53]. The “all-thiol” HMGB1 binds to other chemokines (e.g., CXCL12) and stimulates leukocyte recruitment via the CXCR4 receptor [54] or other signaling molecules [55–57] to the infection or injury sites [58, 59]. In sharp contrast, disulfide HMGB1 can activate immune cells to produce cytokines/chemokines via TLR4 or other receptors, such as RAGE [51], TLR2, TLR4 [60–62], TLR9 [23, 51], cluster of differentiation 24 (CD24)/Siglec-10 [63], Mac-1 [57], thrombomodulin [64], or single transmembrane domain proteins (e.g., syndecans) [65]. Once fully oxidized, HMGB1 is devoid of either chemokine or cytokine activities (Fig. 1) [52, 53]. Altogether, these studies suggest that extracellular HMGB1 is a proinflammatory signal to recruit, alert, and activate innate immune cells, thereby sustaining a potentially injurious inflammatory response during sepsis.

### Pathogenic role of HMGB1 in sepsis and injury

**Fig. 3:**
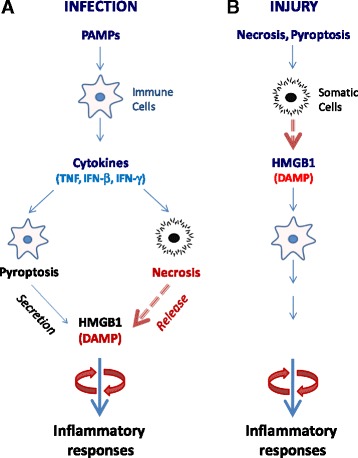
HMGB1 orchestration of infection- and injury-elicited inflammatory responses. **a** A microbial infection triggers a systemic inflammatory response by stimulating active HMGB1 secretion or passive release. The disruption of epithelial barrier allows invasion of microbial pathogens, which liberate PAMPs and trigger the production of proinflammatory cytokines. Several proinflammatory cytokines can stimulate innate immune cells to actively secrete HMGB1 and trigger necroptosis that enables passive HMGB1 release. Collectively, extracellular HMGB1 facilitates leukocyte recruitment and activation, amplifying and sustaining rigorous inflammatory responses. **b** Injury triggers passive HMGB1 release. After injurious insult, HMGB1 is passively released by necrotic cells and functions as a DAMP signal that propagates rigorous inflammatory responses that are indistinguishable from infection-elicited inflammation

Experimental sepsis can be induced by several techniques, including the infusion of exogenous bacterial toxins (endotoxemia) and the disruption of host epithelial barrier, to produce microbial translocation, e.g., cecal ligation and puncture (CLP). In murine models of endotoxemia and CLP-sepsis, HMGB1 is first detected in the circulation 8 h after the disease onset and is subsequently increased to plateau levels from 16 to 32 h (Fig. 3a) [13, 66]. This late appearance of circulating HMGB1 parallels with the onset of animal lethality from endotoxemia or sepsis and distinguishes itself from TNF and other early proinflammatory cytokines [67]. The pathogenic role of HMGB1 in endotoxemia was inferred from studies using HMGB1-neutralizing antibodies, which conferred a dose-dependent protection against endotoxin-induced tissue injury and lethality [13, 68]. In a more clinically relevant CLP-induced sepsis, the delayed administration of HMGB1-specific neutralizing antibodies beginning 24 h *after* CLP dose-dependently rescued rodents from lethal sepsis [32, 66, 69]. Moreover, the targeted inhibition of HMGB1 expression in innate immune cells (e.g., macrophages and dendritic cells) reduces systemic HMGB1 accumulation and similarly rescues mice from sepsis [70], supporting HMGB1 as a critical late mediator of experimental sepsis.

Notably, agents capable of inhibiting HMGB1 release [71–73] or action [13, 66] confer protection against sepsis, particularly if administered in a delayed fashion to strategically preserve the PAMPs-mediated early inflammatory response. At late-stage infection, the PAMPs-mediated inflammatory response may be accompanied by unintended cell injury and DAMPs release that amplify the cytokine storm to precipitate organ dysfunction (Fig. 3a) [29]. This likelihood is supported by recent findings that HMGB1 is persistently elevated during late-stage sepsis despite the cessation of initial infection [74] and that it contributes to the long-term pathological consequence of sepsis. Although microbial infection-induced sepsis is indistinguishable from sterile injury-elicited systemic inflammatory response syndrome [75, 76], it may be more advantageous to develop strategies for specifically attenuating DAMPs-mediated inflammatory responses without compromising the PAMPs-mediated innate immunity.

As a ubiquitous nuclear protein, HMGB1 can be passively released from necrotic cells [37] and can function as a DAMP to elicit inflammatory responses (Fig. 3b). Regardless of the origin, the actively secreted or passively released HMGB1 can similarly alert, recruit, and activate immune cells [49, 77], triggering infection- and injury-elicited systemic inflammatory responses that are often indistinguishable in experimental or clinical settings (Fig. 3) [76]. Indeed, HMGB1-neutralizing antibodies are protective in animal models of ischemia/reperfusion [38, 78, 79], trauma [80, 81], chemical toxemia [42, 82, 83], atherosclerosis [84], gastric ulcer [85] and hyperoxia [86].

### Discovery of Chinese herbs as HMGB1 inhibitors

The establishment of HMGB1 as a mediator of various inflammatory diseases has prompted the search for inhibitors that can attenuate HMGB1 secretion or action in various experimental settings. As summarized in several recent reviews [22, 29, 87], a growing list of herbal extracts (e.g., Danggui, Mung bean, and *Prunella vulgaris*) [88, 89] and components have been demonstrated to be effective in inhibiting endotoxin-induced HMGB1 secretion. In the present review, we compare the distinct mechanisms by which several herbal therapies effectively inhibit active HMGB1 secretion and action.

#### Glycyrrhizin (GZA) binds to HMGB1 to inhibit its secretion or action

**Fig. 4:**
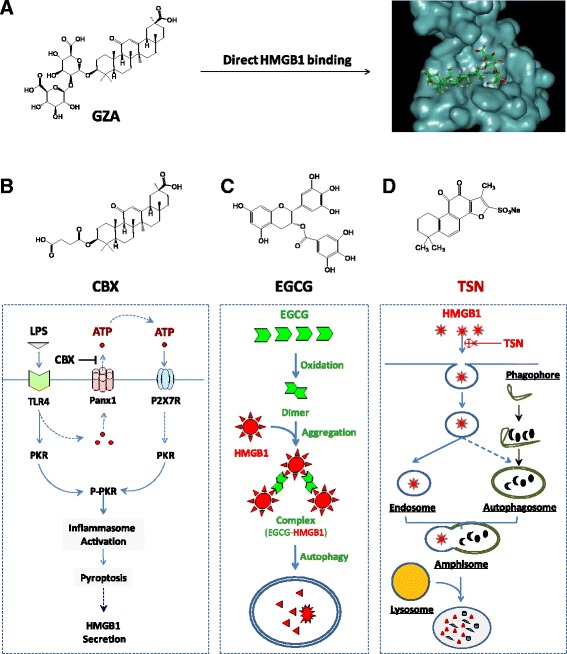
Distinct HMGB1-inhibition mechanisms of several herbal components. **a** Direct binding and inhibition of HMGB1 activities. **b**, **c**, **d** Divergent HMGB1 inhibition mechanisms. Different herbal components can inhibit HMGB1 action or release through divergently distinct mechanisms including PKR inactivation (Panel **b**), autophagic degradation (Panel **c**), or endocytic HMGB1 uptake and degradation (Panel **d**)

*Radix Glycyrrhizae* (*Gancao* in Chinese, meaning “sweet root” in Greek or “licorice” in English) has been traditionally used for treating peptic ulcer, hepatitis, and pulmonary bronchitis for many centuries. Its major anti-inflammatory component, GZA (Fig. 4a), is protective in animal models of hepatitis [90], hepatic ischemia/reperfusion (I/R) injury [91, 92], endotoxin- and acetaminophen-induced liver injury [93, 94]. Using biochemical techniques, Sakamoto et al. (2001) first demonstrated that GZA directly interacted with HMGB1 and impaired its DNA-binding properties [95]. Subsequently, Mollica et al. (2007) used nuclear magnetic resonance (NMR) and fluorescence techniques to confirm that GZA directly docked into the DNA-binding concaves of HMGB1 boxes (Fig. 4a) [96, 97]. Consistent with these findings, the GZA-mediated protection has been associated with the inhibition of HMGB1 release or its cytokine/chemokine activities [87].

#### Carbenoxolone (CBX) prevents PKR activation

Carbenoxolone (CBX) is a chemical derivative of GZA, in which the glucuronic acid is replaced by succinic acid (Fig. 4b). As a medication previously prescribed for esophageal ulceration and inflammation [98], CBX has been demonstrated to dose-dependently inhibit a variety of biological activities, including gap junctions (50–100 μM) and panx1 channels (EC_50_ = 1–4 μΜ) [99, 100]. We recently discovered that CBX effectively inhibited LPS-induced HMGB1 secretion, with estimated IC_50_ at 5 μM and IC_100_ at 10 μM [101]. It appears that CBX effectively inhibited endotoxin-induced HMGB1 release by preventing PKR up-regulation and phosphorylation (Fig. 4b). In light of the findings that CBX (10 μM) could effectively inhibit panx-1-mediated ATP release in response to hypoxia [102], sheer stress [103] and low oxygen tension [104], we propose that crude LPS (*containing trace amounts of bacterial proteins and nucleic acids*) may prime macrophages by up-regulating PKR expression and simultaneously eliciting panx-1-mediated ATP release. The extracellular ATP subsequently binds and activates the purinergic P_2_X_7_ receptor (P_2_X_7_R) [105], which triggers PKR/inflammasome activation and HMGB1 secretion [87, 106].

#### Epigallocatechin-3-gallate (EGCG) stimulates autophagic HMGB1 degradation

Green tea contains a class of biologically active polyphenolic catechins, including the most abundant epigallocatechin-3-gallate (EGCG) (Fig. 4c). At low concentrations, EGCG dose-dependently abrogates LPS-induced HMGB1 secretion, with an estimated IC_50_ < 1.0 μM [72]. Notably, the significant inhibition of HMGB1 secretion is achieved when EGCG is added 2 h to 6 h post LPS stimulation [72], suggesting a likelihood of delayed regimen for EGCG treatment. EGCG appears to prevent LPS-induced HMGB1 secretion strategically by destroying HMGB1 in the cytoplasm via a cellular degradation process, autophagy (“self-eating”) (Fig. 4c).

EGCG can be trafficked into cytoplasmic vesicles (presumably autophagosomes) within 6 h and destined to autophagolysosomes within 16 h [107]. Additionally, EGCG can conjugate to cytoplasmic HMGB1, leading to the formation of EGCG-HMGB1 complexes (dimmer, trimmer, tetramer, and oligomer) (Fig. 4c) [107]. Because these large EGCG-HMGB1 complexes cannot physically pass through the narrow pore of the proteasome barrel of the ubiquitin-proteasome pathway, they trigger the autophagic degradation process. At concentrations effective for inhibiting HMGB1 secretion, EGCG dramatically enhances the formation of autophagosomes [107]. Recently, EGCG has proven to be effective in stimulating autophagy in breast cancer cells [107], hepatocytes [108], retinal pigment epithelial cells [109], and vascular endothelial cells [110]. Given the likelihood that HMGB1 interacts with autophagy regulators (e.g., beclin-1) in the cytoplasm [111, 112], it will be important to investigate whether HMGB1 occupies a critical role in EGCG-mediated autophagy. This assessment is relevant because recent studies have indicated that bacterial endotoxin induces significantly less autophagy in HMGB1-deficient macrophages [19].

#### Tanshinone IIA sodium sulfonate (TSN) stimulates endocytic HMGB1 uptake

*Radix Salviae Miltiorrhizae* (*Danshen* in Chinese) is a medicinal herb that contains several red pigments, including tanshinone I, II, IV, and cryptotanshinone that consist of various anti-inflammatory properties. As a major component (representing 5 % to 6 % of the total dry weight) of Danshen root, tanshinone IIA dose-dependently attenuates LPS-induced HMGB1 secretion, with an estimated IC_50_ < 25 μM. However, its poor water solubility may adversely affect the bioavailability and therapeutic efficacy of tanshinone IIA [73]. One water-soluble derivative, tanshinone IIA sodium sulfonate (TSN) (Fig. 4d), also dose-dependently inhibits LPS-induced HMGB1 secretion with a lower IC_50_ < 10 μM. At doses that completely prevent HMGB1 secretion, TSN does not affect endotoxin-induced release of most other cytokines and chemokines (such as IL-6, IL-12p40/p70, KC, MCP-1, MIP-1α, MIP-2, and TNF), indicating a selectivity for TSN in inhibiting HMGB1 secretion.

Although TSN itself is unable to stimulate autophagic HMGB1 degradation [72], it induces the internalization of exogenous HMGB1 into macrophage cytoplasmic vesicles likely through clathrin- and caveolin-dependent endocytosis (Fig. 4d) [113]. The inhibition of clathrin-dependent (e.g., chlorpromazine) and caveolin-dependent (e.g., nystatin and indomethacin) endocytosis uniformly attenuates the TSN-mediated HMGB1 uptake. Surprisingly, the depletion of several HMGB1 receptors (e.g., TLR2, TLR4, or RAGE) does not impair TSN-mediated enhancement of HMGB1 uptake, suggesting that other HMGB1-binding cell surface proteins (such as, Mac-1, thrombomodulin, or syndecan) may be required for the TSN-mediated HMGB1 internalization.

Intriguingly, emerging evidence has suggested that cytoplasmic HMGB1 is a key activator of autophagy [19, 111, 112], supporting a likely link between TSN-mediated HMGB1 endocytosis and autophagy. When occurring simultaneously, endosomes can fuse with autophagosomes to form amphisomes [114, 115], which merge with lysosomes to form autolysosomes to degrade the amphisome contents [116]. Thus, endocytosis and autophagy can converge on a common lysosome-dependent pathway, leading to eventual HMGB1 degradation. TSN can likely facilitate endocytosis of exogenous HMGB1, leading to the subsequent HMGB1 degradation via a lysosome-dependent pathway (Fig. 4d). It also explains why even when administered several hours after the endotoxin stimulation, TSN can still effectively block HMGB1 secretion.

### Therapeutic efficacy of HMGB1-inhibiting herbs

Current sepsis therapies are largely supportive and limited to a few clinical interventions, including antibiotics, steroidal anti-inflammatory drugs (e.g., hydrocortisone) and early goal-directed therapies (EGDT). For instance, appropriate broad-spectrum antibiotics are often administered to patients to facilitate the elimination of bacterial pathogens [2]; however, the release of bacterial products (e.g., endotoxin or CpG-DNA) may adversely amplify inflammatory responses. Accordingly, anti-inflammatory steroids (e.g., hydrocortisone, methylprednisolone, dexamethasone, and fludrocortisone) are frequently used to modulate the excessive inflammatory response, despite the lack of reproducible efficacy in clinical sepsis trials [117–119]. As a supportive intervention, EGDT employs extremely tight control of numerous physiological parameters (such as central venous pressure, mean arterial blood pressure, central venous oxygen saturation, and hematocrit) with discrete, protocol-driven interventions of crystalloid fluids, vasopressors, and blood transfusions. Unfortunately, this simple intervention was ineffective in reducing septic mortality [120, 121], prompting the search for other HMGB1-targeting agents for treating sepsis in humans.

Given that various herbal components are capable of preventing endotoxin-induced HMGB1 secretion, we explored their efficacy in animal models of CLP-induced sepsis. Considering the late and prolonged kinetics of HMGB1 accumulation in experimental sepsis [66], the first dose of HMGB1 inhibitors was administered in a delayed fashion 24 h after the onset of sepsis. Repetitive intraperitoneal administrations of EGCG [72], TSN [73], or CBX [101] at 24 h, 48 h, and 72 h post CLP significantly increased animal survival rates. When administered orally, EGCG rescued mice from lethal sepsis, significantly increasing animal survival rates from 16 % to 44 % [107]. Intriguingly, we found that EGCG facilitated bacterial elimination in selective organs (e.g., liver and lung) in an animal model of sepsis [122]. Importantly, these herbal components have demonstrated to be beneficial in other models of inflammation, such as ischemia trauma, crush injury, radiation, and chemical toxemia [87]. It is not yet known whether these protective effects are associated with the inhibition of HMGB1 release or chemokine/cytokine activities.

Recently, an herbal remedy consisting of five herbs (*Radix Angelicae Sinensis* (*Danggui* in Chinese), *Radix Salviae Miltiorrhizae* (*Danshen* in Chinese), *Flos Carthami* (*Honghua* in Chinese), *Rhizoma Ligustici Chuanxiong* (*Chuanxiong* in Chinese), and *Radix Paeoniae Rubra* (*Chishao* in Chinese)) has been developed in China for treating septic patients. This combinational therapy, termed *Xuebijing* (name in Chinese) has proven to be protective in animal models of sepsis [123] or in patients with sepsis [124, 125]. Considering the distinct but likely complementary mechanisms, HMGB1 inhibition and other combinational therapy might also be associated with improved therapeutic efficacy. For instance, the induction of autophagy by EGCG may provide a negative feedback regulation of inflammasome activation by eliminating damaged mitochondria [126], removing active inflammasomes [126, 127], and destroying cytoplasmic HMGB1 [107]. It is thus important to test whether improved protection could be achieved by combinational therapy using HMGB1 inhibitors that divergently modulate autophagy (e.g., EGCG) and inflammasome (e.g., CBX). These important studies may pave the road to future clinical studies that explore the therapeutic potential of additional herbal cocktails for treating sepsis and other inflammatory diseases.

## Conclusions and outlook

For complex systemic inflammatory syndromes, it is difficult to translate successful animal studies into clinical applications, in part, because of the pitfalls in the selection of non-feasible therapeutic targets or non-realistic clinical outcome measures, such as survival rates [1]. For instance, therapeutic strategies targeting PAMPs (e.g., endotoxin) [128] or PAMP signaling (e.g., eritoran) [129] fail to improve survival in clinical trials of human sepsis, raising questions regarding the feasibility of PAMPs-blocking agents in the treatment of infectious diseases. However, the investigation of pathogenic cytokines in animal models of diseases has led to the development of successful cytokine-targeting therapeutic strategies (e.g., chimeric anti-TNF monoclonal antibody, infliximab, and a soluble TNF receptors-Fc fusion protein, sTNF-R-Fc, etanercept) for autoimmune diseases, such as rheumatoid arthritis [130]. Thus, there is ongoing research for other clinically feasible therapeutic targets (such as IL-3) and medications for human sepsis [131].

HMGB1, which is secreted from immunologically activated innate immune cells and is released from pathologically damaged cells, functions as a critically important mediator in lethal infection and injury. In animal models of sepsis, HMGB1-neutralizing antibodies or inhibitors can rescue mice from the lethality, particularly if administered in a delayed manner to preserve the potentially beneficial early PAMPs-mediated inflammatory responses [132]. Developing novel strategies for specifically modulating DAMP-elicited injurious inflammatory response without impairing the PAMP-mediated beneficial innate immunity against infection may be possible. Future clinical studies are anticipated to test the efficacy of HMGB1-neutralizing antibodies in the clinical management of human inflammatory diseases.

However, humanized monoclonal antibodies (mAb) are manufactured in low-yield and time-consuming mammalian cells and are thus more expensive than small molecule chemical agents [29]. It is thus essential to develop cost-effective, small molecule agents for the clinical management of human sepsis. One of the most selective HMGB1 inhibitor, TSN, has already been used in China as medication for patients with cardiovascular disorders. The capacity to facilitate endocytic HMGB1 uptake by professional phagocytes may provide the basis for the treatment of both infection- and injury-elicited inflammatory diseases [29]. It is not yet known whether better protection could be achieved by a combinational therapy with several anti-HMGB1 agents. It is thus important to explore the therapeutic potential of these HMGB1-inhibiting agents in future studies.

## Abbreviations

CBX: Carbenoxolone
CIRP: Cold-inducible RNA-binding protein
CLP: Cecal ligation and puncture
DAMP: Damage-associated molecule pattern molecules
EGCG: Epigallocatechin-3-gallate
GZA: Glycyrrhizin
HMGB1: High mobility group box 1
IFN: Interferon
IL: Interleukin
IC_50_: The half maximal inhibitory concentration
LPS: Lipopolysaccharide
NLS: nuclear localization sequence
NLR: Nod-like receptor
PAMP: Pathogen-associated molecular pattern molecule
PKR: Double-stranded RNA-activated protein kinase R
RAGE: Receptor for advanced glycation end products
RIP: Receptor interacting protein
STAT1: Signal transducer and activator of transcription 1
TLR: Toll-like receptor
TNF: Tumor necrosis factor
TSN: Tanshinone IIA sodium sulfonate
